# Transcriptional ontogeny of first trimester human fetal and placental mesenchymal stem cells: Gestational age versus niche

**DOI:** 10.1016/j.gdata.2014.10.016

**Published:** 2014-10-25

**Authors:** Jennifer M. Ryan, Nicholas Matigian, Rebecca A. Pelekanos, Samuel Jesuadian, Christine A. Wells, Nicholas M. Fisk

**Affiliations:** a The University of Queensland, UQ Centre for Clinical Research, Herston Campus, Herston QLD 4029, Australia; b Australian Institute for Bioengineering and Nanotechnology, The University of Queensland, Brisbane St. Lucia, QLD 4072, Australia; c Centre for Advanced Prenatal Care, Royal Brisbane & Women's Hospital, Herston QLD 4029, Australia; d Eskitis Institute for Cell and Molecular Therapies, National Centre for Adult Stem Cell Research, Griffith University, Brisbane, Australia; e The Institute for Infection, Immunity and Inflammation, College of Medical, Veterinary and Life Sciences, The University of Glasgow, Scotland G12 8TA, UK

**Keywords:** Fetal, Placental, Mesenchymal stem/stromal cells, First trimester, Gestational age

## Abstract

Early fetal and placental MSCs have translationally-advantageous characteristics compared to later pregnancy MSCs. During the first trimester, the fetus and placenta grow rapidly with divergent developmental requirements, but studies comparing mesenchymal stem cells (MSCs) from different origins have paid little attention to the effect of gestational age over this temporal window. Here we present the transcriptome through first trimester development of MSC isolated from fetal bone marrow (BM) or placental structures. Samples were collected weekly from 8 to 12 weeks. The raw microarray data are available on the ArrayExpress database (www.ebi.ac.uk/arrayexpress) under accession number E-TABM-1224. Additionally, the data have been integrated into the stem cell collaboration platform www.Stemformatics.org. These data provide a valuable resource for developmental biology and stem cell investigation.

**Table:** 

Specifications	
Organism/cell line/tissue	Human
Sex	Male & female
Sequencer or array type	Illumina HumanHT-12 v4 BeadChip
Data format	Raw and normalized
Experimental factors	Tissues and gestational age comparison
Experimental features	Transcriptome of fetal bone marrow (BM) or placental MSCs through first trimester
Consent	Ethically-approved sample collection and processing. Anonymised results.
Sample source location	Brisbane, Australia

## Direct link to deposited data

http://www.ebi.ac.uk/arrayexpress/experiments/E-TABM-1224/

http://www.stemformatics.org/datasets/view/6063

## Experimental design, materials and methods

### Sample collection

The Human Research Ethics Committees of the Royal Brisbane and Women's Hospital and the University of Queensland approved the tissue collection. Women gave written informed consent for the use of fetal tissue for research purposes after clinically-indicated termination of pregnancy in compliance with national research guidelines.

Fetal MSC from bone marrow (BM) and placental tissues [chorionic membrane (CM), and chorionic villi (CV)] were obtained from three donors at five time points across the first trimester (8, 9, 10, 11 and 12 weeks). Developmental age was expressed in gestational weeks, as per convention and as used clinically (postconceptual age plus two weeks). Gestational age of the fetal and placental tissues collected was determined by an ultrasound (Sonoscope 3.5–4.5 MHz), using a crown rump length between 7 and 11 weeks, and biparietal diameter thereafter, expressed in days and rounded to the nearest half week.

### Stem cell isolation and culture

Fetal BM-MSCs were prepared by flushing fetal long bones using a 21-gauge syringe [1]. Single cell suspensions of fetal BM were washed and filtered through a 70 μm nylon filter into Dulbecco's modified Eagle's medium (DMEM) (Invitrogen). MSCs from CM and CV were isolated by enzymatic digestion with dispase (2.4 U/ml) and collagenase (240 U/ml) using protocols adapted from Steigman et al. 2007 [2]. MSCs were then cultured in DMEM supplemented with 10% fetal bovine serum, antibiotic and antimycotic solution (100×, Invitrogen), at 37 °C with 5% CO_2_. Isolated MSCs were characterized by a typical cell surface phenotype and differentiation capacity. All MSCs used were between passages 1 and 6.

### Differentiation assays

Differentiation potential of each MSC source was performed according to the previously published methods [3]. Staining for both adipogenic and osteogenic assays was visualized using a bright field phase contrast microscopy.

For adipogenesis, 2 × 10^5^ MSCs were seeded in 6 well plates until confluent, and differentiation induced by culturing the cells in adipogenic media containing; indomethacin (60 μM), dexamethasone (1 μM), insulin (5 μg/ml) and isobutylmethylxanthine (IBMX) (0.5 mM). All reagents were from Sigma-Aldrich. After 21 days, cells were fixed with 4% paraformaldehyde with PBS and stained with Oil Red O. Adipogenic differentiation was determined by the appearance of Oil Red O indicating the formation of lipid vacuoles, characteristic of adipogenic cells.

Osteogenic differentiation was induced by culturing 3 × 10^5^ cells in 6 well plates in the presence of osteogenic induction media containing dexamethasone (0.1 μM), β-glycerol phosphate (10 mM) and l-ascorbate-2-phosphate (0.2 mM) for 21 days. All reagents were from Sigma-Aldrich. Cells were fixed in 4% paraformaldehyde in PBS and stained with Alizarin red. Differentiation was determined by the appearance of red deposits, representing areas of mineralized calcium.

### Flow cytometry

All MSCs were immunophenotyped by flow cytometry [3]. MSCs were washed and resuspended in PBS containing 2% FBS. 100 μl of cells (1 × 10^6^) were incubated with cell surface antigen CD11b-APC, CD14-Pe/Cy5, CD29-Pe, CD31-APC, CD34-Pe, CD44-FITC, CD45-Pe/Cy5, CD73-APC, CD73-APC, CD90-FITC and CD105-FITC or the appropriate isotype controls (eBiosciences or BD Biosciences) for 20 mins at 4 °C. Cells were acquired using Gallios Flow Cytometer and analyzed using Kaluza software (Beckman Coulter).

### T cell proliferation assay

The mitogen-driven T cell proliferation assay was performed by stimulating PBMC with 1 μg/ml Phytohemagglutinin (PHA) in flat-bottom 96-well plates (Nunc) in a total of 200 μM RPMI 1640 supplemented with 10% heat-inactivated FCS and 1% penicillin/streptomycin (Invitrogen). Cells were cultured as follows: 2 × 10^5^ per well PBMC were cultured with or without 1.5 × 10^4^ MSC in the presence of 0.1 μg/ml PHA and PBMC. After 4 days in culture, 1 μCi ^3^H-Thymidine was added to each well and samples were incubated for further 6 h at 37 °C in a humidified CO_2_ incubator. Cells were then harvested onto glass fiber filter mats using a Skatron harvester. ^3^H-Thymidine incorporation was measured using a β-plate scintillation counter. Proliferation was represented as the incorporated radioactivity in counts per minute (CPM). Results are expressed as the mean of triplicate values ± SE.

### MSC phenotyping

**Fig. 1:**
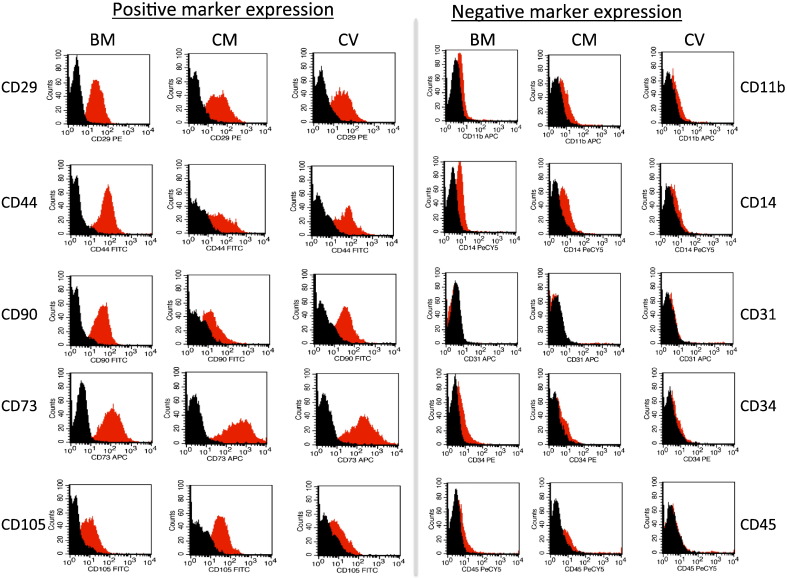
Immunophenotyping of fetal bone marrow (BM), chorionic membrane (CM) and chorionic villi (CV) by flow cytometric analysis. Representative histograms demonstrating positive and negative expression of surface proteins. Similar expression levels were observed for each source. The cells were positive for CD29, CD44, CD90, CD73 and negative for CD11b, CD14, CD31, CD34 and CD45. Red Peak represents marker of interested, black peak represents isotype control.

Human MSCs from fetal BM, placental CM, and placental CV were isolated at 8, 9, 10, 11 and 12 weeks. Universally, each MSC source met the criteria set by the International Society for Cellular Therapy [4] being plastic adherent, fibroblastic in morphology and expressed the various MSC markers. Using flow cytometry, cell surface marker expression for each source and gestational age was shown to be similar, indicating the immunophenotype was not affected by gestational age or MSC source. MSCs expressed CD29, CD44, CD73, CD90 and CD105 and were negative for hematopoietic and endothelial cell markers (CD14, CD34, CD45 and CD31) (Fig. 1).

**Fig. 2:**
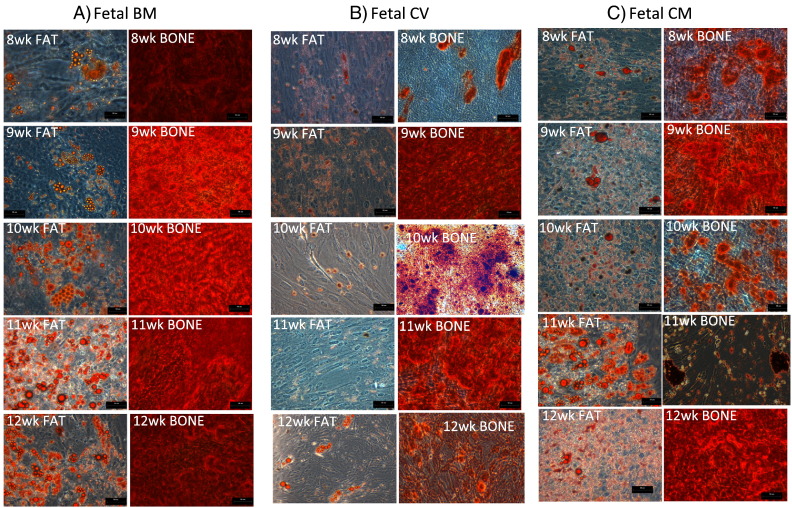
Representative images of multi-differentiation potential of first trimester fetal bone marrow (BM), chorionic villi (CV) and chorionic membrane (CM) at 8 –12 weeks. Adipogenesis was determined using oil red O staining of lipid droplets after 21 days in adipogenic media, and osteogenesis using alizarin red staining for mineral matrix deposition after 21 days in osteogenic media. Scale bar = 50 μM.

Each MSC source and gestation was examined for its ability to differentiate into adipocytes and osteocytes under appropriate inductive culture conditions. Results showed some variability between MSC sources. BM-MSC at each gestation were consistently superior at differentiating into osteocytes compared to CV-MSC and CM-MSC. Early BM-MSC and CM-MSC (8 & 9 weeks) were considerably slower to undergo adipogenesis compared to later gestations (10–12) and CV-MSC were particularly poor at adipogenic differentiation at each gestational age examined (Fig. 2).

**Fig. 3:**
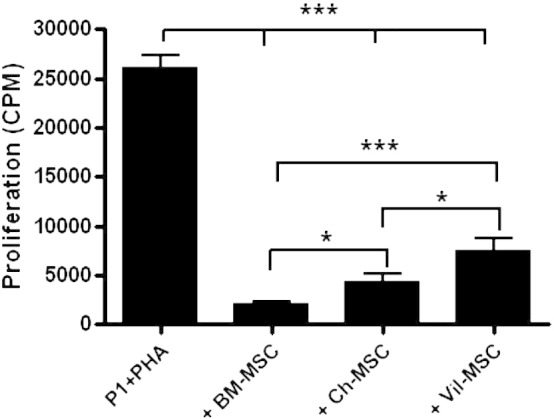
T cell proliferation was reduced by BM-MSC compared to CM-MSC (*, *p* = 0.0161) and CV-MSC (***, *p* = 0.0002). CM-MSC showed greater ability to suppress proliferation compared to CV-MSC (*, *p* = 0.0371). Expressed as mean CPM +/− SE.

To investigate the functional immunosuppressive properties of fetal-MSC and placental MSC, in vitro mitogen-driven proliferation assays were used to evaluate their ability to suppress T cell proliferation. PHA was used as a standard mitogen to trigger proliferation of lymphocytes. The effect of MSC on T cell proliferation was evaluated by incubating PBMC with either BM-MSC, CM-MSC or CV-MSC. As expected, all MSC sources suppressed mitogen-driven proliferation of PBMC, however, BM-MSC showed a greater reduction of PBMC proliferation compared to CM-MSC (*p* = 0.0161) and CV-MSC (*p* = 0.0002), while CM-MSC showed a greater ability to suppress proliferation compared to CV-MSC (*p* = 0.0371) (Fig. 3).

### RNA extraction

Total RNA was extracted from all the MSC samples using the RNeasy Mini Kit (Qiagen, Hilden, Germany) according to the manufacturer's instructions. Total RNA yield was determined using Nanodrop 1000, and the quantity and quality were validated using the Agilent 2100 Bioanalyzer (Agilent Technologies).

### Illumina HumanHT-12 v4 BeadChip microarray

To determine whether there was a niche or gestational effect on MSC, we analyzed the transcriptome profiles of three MSC sources: bone marrow (BM)-MSC, chorionic membrane (CM)-MSC and chorionic villi (CV)-MSC using the Illumina HumanHT-12 v4 BeadChip array (Illumina — BD-103-0204 San Diego, CA 92122 USA). MSC isolated from bone marrow (BM)-MSC, chorionic membrane (CM)-MSC and chorionic villi (CV)-MSC were studied longitudinally over 5 weeks (8–12 weeks gestation). We analyzed three donors at each gestational age from each source (n = 45 total MSC donors).

Briefly, total RNA (500 ng) was converted to biotinylated cRNA using Illumina TotalPrep RNA Amplification Kit (Invitrogen — AMIL1791) (as per proprietary instructions) and hybridized to HumanHT-12 v4 BeadChip (Illumina — BD-103-0204). The hybridized BeadChip was washed and scanned with the Illumina BeadStation System.

### Microarray data

**Fig. 4:**
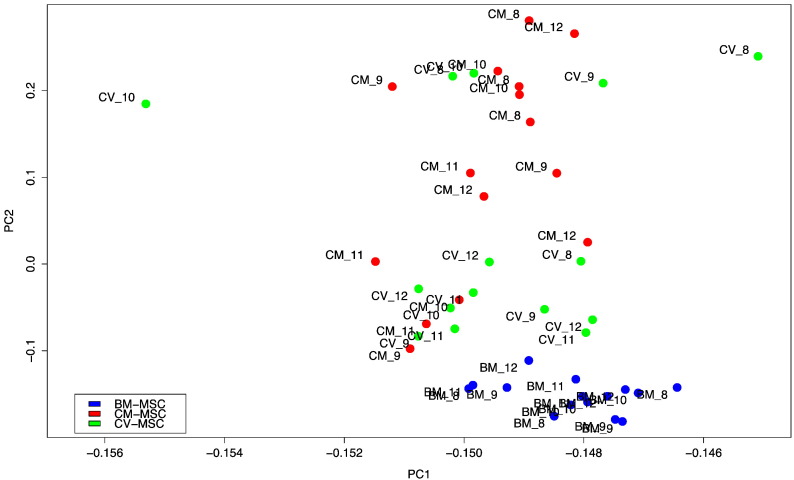
Principal Component Analysis (detected probes only). Components 1 (93.2% variance) and 2 (2.1% variance); Legend: MSC source indicated by color: bone marrow (BM) (blue); chorionic membrane (CM) (red); chorionic villi (CV) (green). Label number indicates gestational stage (weeks).

Raw data were processed using GenomeStudio version 2011.1 and exported with no preprocessing. Background correction and normalization was performed using the Bioconductor package ‘lumi’ [5]. Microarray data were deposited into ArrayExpress (Accession Number E-TABM-1224). Principal Component Analysis is shown in Fig. 4. Additionally, the data have been integrated into a public portal, Stemformatics [6]. Here all the microarray data can be visualized and compared with 100 + other stem cell datasets (http://www.stemformatics.org).

## Author contributions

NF conceived the idea, JR and SJ collected samples, and JR with SJ characterized MSC including immunosuppressive assay. NM and CW performed and analyzed the micro-array and JR, RP, and NF assisted in data interpretation. JR, NM, CW and NF wrote the paper, and all the authors contributed to the drafts and have read and approved the final manuscript.
